# Exploring the relationship between mental health status and life events in children and adolescents: the role of mediating factors

**DOI:** 10.3389/fpsyt.2025.1520353

**Published:** 2025-02-04

**Authors:** Jiemei Xie, Guoqiang Cheng, Mingliang Zhou, Qisheng Liang, Qianjin Wang

**Affiliations:** ^1^ Department of Psychiatry, Zhaoqing Third People’s Hospital, Zhaoqing, Guangdong, China; ^2^ Department of Psychiatry, Shantou University, Shantou, Guangdong, China; ^3^ Department of Psychiatry, Shandong Provincial Hospital Affiliated to Shandong First Medical University, Jinan, Shandong, China; ^4^ Department of Psychiatry, Shandong Provincial Hospital, Shandong University, Jinan, Shandong, China; ^5^ Department of Psychiatry, National Clinical Research Center for Mental Disorders, and National Center for Mental Disorders, The Second Xiangya Hospital of Central South University, Changsha, Hunan, China

**Keywords:** negative life events, depressive symptoms, anxiety symptoms, children and adolescents, mediation analysis

## Abstract

**Background:**

The mental health status of children and adolescents is significantly influenced by negative life events, yet there is a lack of comprehensive analysis on mediating factors. This study aims to examine the relationship between negative life events and symptoms of anxiety and depression in children and adolescents, as well as to identify potential mediating factors.

**Methods:**

In May 2022, cluster-randomized sampling was used to select 8041 primary, middle, and high school students in Zhaoqing City, Guangdong Province, for the final analysis. The Adolescent Life Events Scale, Self-Rating Anxiety Scale, and Self-Rating Depression Scale were employed to assess negative life events, anxiety symptoms, and depressive symptoms, respectively. Pearson correlation analysis and linear regression analysis were utilized to identify risk factors, and the Bootstrap method was applied for mediation analysis.

**Results:**

The study revealed that 80.52% of children and adolescents experienced negative life events, with 23.38% reporting anxiety symptoms and 42.57% reporting depressive symptoms. Significant positive correlations were observed between negative life events and anxiety symptoms (r = 0.32, *p* < 0.001), as well as depressive symptoms (r = 0.44, *p* < 0.001). Mediation analysis further demonstrated that anxiety and depressive symptoms acted as mutual mediators in the relationship between negative life events and each other.

**Conclusions:**

This study underscores the complex interplay between anxiety, depression, and negative life events in children and adolescents. These findings can inform strategies to help mitigate the emotional impact of negative life events, enhance mental health, and provide a scientific basis for developing effective interventions.

## 1 Introduction

Mental health problems are highly prevalent among children and adolescents, with the most common issues being negative emotional states such as depressive and anxiety symptoms (1). Negative life events in these age groups are significant risk factors for mental health issues, as demonstrated by various studies exploring the correlation between mental health problems and negative life events (2, 3). According to DeLongis et al., major life events act as distal influences on physical and mental health, while daily life events serve as proximal influencing factors (4). Large-scale public health crises, such as the COVID-19 pandemic, have further intensified anxiety and depressive symptoms in children and adolescents due to widespread social and lifestyle disruptions, profoundly affecting their overall well-being (5). Moreover, chronic anxiety and depression during these formative years are strong predictors of persistent mental health challenges in adulthood (6). These findings underscore the critical importance of prioritizing mental health interventions for children and adolescents.

Negative life events are significant contributors to the development of anxiety and depression in children and adolescents (7). Research consistently shows a strong association between negative life events and the onset of these mental health issues (8). Such events often serve as direct triggers for depressive symptoms (9). For example, children and adolescents who encounter negative life events—such as challenges related to relationships, academic pressures, health issues, and adjustment difficulties—are at an increased risk of developing severe depressive symptoms. Furthermore, depressive symptoms themselves can predict the occurrence of additional negative life events (10, 11), creating a vicious cycle where initial depressive symptoms amplify the likelihood of subsequent negative life events, which in turn worsen the symptoms (12). Studies have highlighted this bidirectional relationship: the frequency and severity of negative life events correlate with higher levels of depressive symptoms, and vice versa (13, 14), suggesting a pattern of mutual causation. Negative life events are also closely linked to anxiety disorders (15), with anxiety symptoms frequently emerging in children and adolescents following such experiences (16). While the mechanisms underlying this association are less well-studied, several investigations suggest that life events often precede the onset of anxiety disorders (17).

Most existing studies focus on the relationship between negative life events and psychological problems in children and adolescents (18–20), while analysis of potential mediating factors is clearly insufficient. As a result, the relationship between negative life events and depressive and anxiety symptoms in healthy children and adolescents remains underexplored. To better understand how anxiety, depression, and life events are interconnected, the fields of behavioral medicine and psychology often involve constructing multiple mediating variables to clarify the mechanisms through which independent variables influence dependent ones (21). This approach helps reduce estimation errors that may arise when mediating factors are overlooked. Therefore, this study hypothesizes that there is a reciprocal influence between anxiety symptoms, depressive symptoms, and negative life events, with potential underlying mediating factors. The aim of this study is to explore the interactions between depressive and anxiety symptoms and negative life events in children and adolescents, as well as the mediating factors involved, in order to provide a more comprehensive foundation for both theoretical understanding and practical interventions, ultimately contributing to the improvement of mental health outcomes in this population.

## 2 Materials and methods

### 2.1 Participants and procedures

The cross-sectional investigation method was used to study the primary, middle and high school students in Zhaoqing city, Guangdong province. The whole random sampling method was adopted, and the questionnaire was distributed uniformly by the investigator to guide the students to complete the survey. A total of 8,245 questionnaires were distributed, of which 204 were excluded due to incomplete information, multiple or missing selections, or other invalid questionnaires, with a valid response rate of 97.5%. They were all between 7 and 20 years old.

### 2.2 Sociodemographic information

Socio-demographic characteristics, including age, sex, and educational level, were collected by self-designed questionnaires.

### 2.3 Measures

#### 2.3.1 Negative life events

The Adolescent Life Events Scale was used to assess the physical and mental health of adolescents based on their physiological and psychological characteristics and social roles in Chinese society (22). This scale has been validated through extensive testing (23, 24), demonstrating robust reliability and validity. The scale comprises 27 items across six factors: interpersonal relationships, academic stress, punishment, loss, health adaptation, and others. Responses are scored on a 5-point Likert scale: “1” indicates no impact, “2” mild impact, “3” moderate impact, “4” severe impact, and “5” extreme impact. Higher scores reflect greater stress experienced by the individual.

#### 2.3.2 Anxiety symptoms

The Self-Rating Anxiety Scale (SAS) was developed to assess anxiety status of adolescents (25). This scale consists of 20 items, each rated on a 4-point scale: “1” indicates no or very little time, “2” indicates some of the time, “3” indicates most of the time, and “4” indicates most or all the time. It evaluates the subjective feelings of respondents over the past week, with higher scores indicating a higher tendency toward anxiety.

#### 2.3.3 Depressive symptoms

The Self-Rating Depression Scale (SDS) was used to evaluate the depressive status of adolescents (26). This scale includes 20 items, rated on a 4-point scale: “1” represents no or very little time, “2” represents a small amount of time, “3” represents a considerable amount of time, and “4” represents most or all the time. It assesses the respondents’ subjective feelings over the past week, with higher scores indicating a greater tendency toward depression.

### 2.4 Statistical analysis

Data were analyzed using SPSS 26.0. Measurement data conforming to a normal distribution were expressed as mean ± standard deviation (M ± SD). Comparisons between groups were conducted using the t-test. The variance inflation factor (VIF) was used to assess multicollinearity among independent variables, with VIF > 5 indicating significant multicollinearity. Spearman correlation analysis was conducted to examine the relationships among life events, anxiety, and depression in children and adolescents. Multiple linear regression was used to analyze these relationships further. The Bootstrap method was employed to test the significance of the mediating effects among life events, anxiety, and depression. A total of 5000 bootstrap samples were drawn, and the 95% bootstrap confidence interval (CI) was calculated. A 95% CI that did not include zero indicated a significant mediating effect (27). A *p*-value < 0.05 was considered statistically significant (two-tailed).

## 3 Results

### 3.1 Sociodemographic and clinical characteristics

As outlined in **Table 1**, the sociodemographic and clinical characteristics of the 8041 children and adolescents included in the study are as follows: The age distribution consisted of 4364 children aged ≤13 years (54.3%), 3592 children aged 13-18 years (44.7%), and 85 children aged ≥18 years (1.1%). The gender distribution showed 4344 males (54.3%) and 3697 females (46.0%). In terms of education level, 2719 participants (33.8%) had primary school education, 3850 participants (47.9%) had middle school education, and 1472 participants (18.3%) had high school education.

**Table 1:** Sociodemographic and clinical characteristics of adolescents.

Variables	Overall numbers (n = 8041)
Age, (n, %)	
13 or less	4364 (54.3%)
13 to 18	3592 (44.7%)
18 or more	85 (1.1%)
Sex, (n, %)	
male	4344 (54.0%)
female	3697 (46.0%)
Educational level, (n, %)	
primary school	2719 (33.8%)
junior high school	3850 (47.9%)
senior high school	1472 (18.3%)
SAS scores, (M ± SD)	43.42 ± 10.07
SDS scores, (M ± SD)	51.09 ± 10.90
ASLEC scores, (M ± SD)	45.25 ± 16.02
interpersonal relationship	9.41 ± 3.92
study pressure	8.84 ± 3.48
punishment	10.80 ± 4.76
bereavement	4.78 ± 2.60
healthy adaptation	5.43 ± 2.09
other factors	6.00 ± 2.55

M, Mean; SD, standard deviation; n, number; %, percentage; SAS, Zung's Self-Rating Anxiety Scale; SDS, Zung's Self-Rating Depression Scale; ASLEC, Adolescent Self-Rating Life Events Check List.

The analysis of 8,041 participants reveals that 1,880 (23.38%) experienced mild or higher levels of anxiety, 6,475 (80.52%) reported a total life events score above the cutoff of 32, indicating negative life events, and 3,423 (42.57%) showed mild or higher levels of depression. Among primary school students, 23.28% experienced anxiety, 74.55% reported significant negative life events, and 45.75% had depressive symptoms. In middle school students, these proportions were 23.74%, 80.91%, and 43.12%, respectively. For high school students, 22.62% experienced anxiety, 90.56% reported significant negative life events, and 35.26% showed depressive symptoms.

### 3.2 Univariate analysis of SDS scores

**Table 2** demonstrates that there were significant differences in SDS scores among different age (t = 55.91, *p* < 0.001), gender (t = 55.91, *p* < 0.001), and education levels (t = 3.30, p < 0.001).

**Table 2:** Univariate analysis of SDS scores in adolescents (N = 8041).

	SDS scores (IQR)	Test statistics	*p*
Age		55.91	< 0.001
13 or less	52.5 (43.8, 60.0)		
13 to 18	50.0 (42.5, 57.5)		
18 or more	51.3 (43.8, 57.5)		
Sex^#^		3.30	< 0.001
male	50.0 (42.5, 57.5)		
female	51.3 (43.8, 60.0)		
Educational level		42.50	< 0.001
primary school	51.3 (43.8, 60.0)		
junior high school	51.3 (42.5, 58.8)		
senior high school	48.8 (42.5, 56.3)		

IQR, interquartile range; SDS, Zung's Self-Rating Depression Scale. # means a two-sample Kolmogorov-Smirnov Test was performed. All *p*-values survived Bonferroni correction (*p* < 0.05/8 = 0.004).

### 3.3 Results of the correlation analysis

According to **Table 3**, depressive symptoms were found to be positively correlated with anxiety symptoms (r = 0.67, *p* < 0.001) and adolescent life events (r = 0.32, *p* < 0.001). Conversely, it was negatively correlated with age (r = -0.08, *p* < 0.001), gender (r = -0.08, *p* < 0.001), and education level (r = -0.07, *p* < 0.001). Anxiety symptoms exhibited a positive correlation with adolescent life events (r = 0.44, *p* < 0.001) and a negative correlation with gender (r = -0.09, *p* < 0.001), with no statistically significant correlation with age and education level. Adolescent life events were positively correlated with age (r = 0.07, *p* < 0.001) and education level (r = 0.11, *p* < 0.001), but negatively correlated with gender (r = -0.06, *p* < 0.001). There was also a positive correlation between age and education level (r = 0.73, *p* < 0.001), and no statistically significant correlation between gender and education level.

**Table 3:** Spearman correlations between anxiety symptoms, depressive symptoms, and negative life events in adolescents.

	1	2	3	4	5	6
1 SDS scores	1					
2 SAS scores	0.67^***^	1				
3 ASLEC scores	0.32^***^	0.44^***^	1			
4 Age	-0.08^***^	-0.02	0.07^***^	1		
5 Sex	-0.08^***^	-0.09^***^	-0.06^***^	-0.006	1	
6 Educational level	-0.07^***^	0.003	0.11^***^	0.73^***^	-0.02	1

SAS, Zung's Self-Rating Anxiety Scale; SDS, Zung's Self-Rating Depression Scale; ASLEC, Adolescent Self-Rating Life Events Check List. ***: < 0.001. All *p*-values survived Bonferroni correction (*p <*0.05/36 = 0.0014).

### 3.4 Stepwise linear regression analysis

The results presented in **Table 4** indicate a significant positive correlation between depressive symptoms and anxiety symptoms (β = 0.66, *p* < 0.0001). Additionally, a significant negative correlation was observed between adolescent depressive symptoms and negative life events (β = -0.05, *p* < 0.0001). Depressive symptoms also showed a positive correlation with education level (β = 0.05, *p* < 0.0001).

**Table 4:** Stepwise linear regression results for depressive symptoms in adolescents.

	B	SE	Beta	t	p	VIF	R^2^	Adjusted R^2^
SAS scores	0.71	0.01	0.66	71.32	< 0.0001	1.29	0.47	0.47
ASLEC scores	-0.73	0.18	-0.05	-3.98	< 0.0001	2.11		
Educational level	0.03	0.006	0.05	5.18	< 0.0001	1.28		
Sex	-0.54	0.18	-0.03	-3.01	0.003	1.01		
Age	-0.63	0.25	-0.03	-2.56	0.01	2.11		

SE, Standard Error; VIF, Variance Inflation Factor; SAS, Zung's Self-Rating Anxiety Scale; ASLEC, Adolescent Self-Rating Life Events Check List.

### 3.5 Results of the bootstrap analysis of mediation effects

To gain a deeper understanding of mediating effects, this study assessed the significance of each indirect effect by applying the bootstrap procedure, which would involve plotting 5000 bootstrap samples and calculating 95% CI. The 95% CI for each pathway excluded zero, indicating significant overall, direct, and indirect effects.

**Figure 1:**
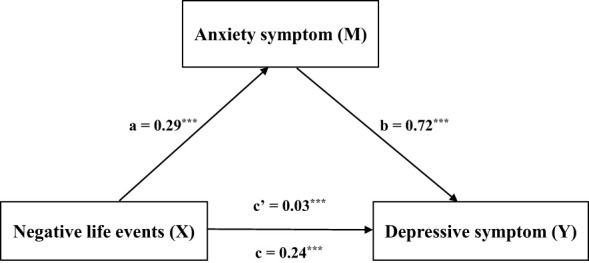
Visually illustrates this mediating relationship, reinforcing the importance of anxiety symptoms in the connection between negative life events and depressive symptoms. Mediating results of anxiety symptoms, depression symptoms, and negative life events (N = 8041). The results showed that anxiety symptoms, depression symptoms, and negative life events in children and adolescents were the mediating factors of the relationship between the other two variables, that is, mutual mediating factors. ***p < 0.001.

**Table 5** shows that the overall effect of negative life events on depressive symptoms was 0.24 (SE = 0.007, 95% CI = 0.22-0.25, t = 33.21, *p* < 0.001). The direct effect was 0.03, accounting for 12.5% (SE = 0.006, 95% CI = 0.01-0.04, t = 4.58, *p* < 0.001). The indirect effect was 0.21, accounting for 87.5% (SE = 0.006, 95% CI = 0.02-0.22). **Figure 1** visually illustrates this mediating relationship, reinforcing the importance of anxiety symptoms in the connection between negative life events and depressive symptoms.

**Table 5:** Total, direct, and indirect effects of negative life events (X) on depressive symptoms (Y) through anxiety symptoms (M) (N = 8041).

Effect	Estimate	Boot SE	Total Effect Percentage	Boot 95% CI		t	*p*
				Lower	Upper		
Total effect	0.24	0.007	100%	0.22	0.25	33.21	< 0.001
Direct effect	0.03	0.006	12.5%	0.01	0.04	4.58	< 0.001
Indirect effect	0.21	0.006	87.5%	0.20	0.22	–	–

SE, Standard Error; CI, confidence interval. Based on 5000 bootstrap samples; 95 % Bias Corrected Confidence Interval.

**Figure 2:**
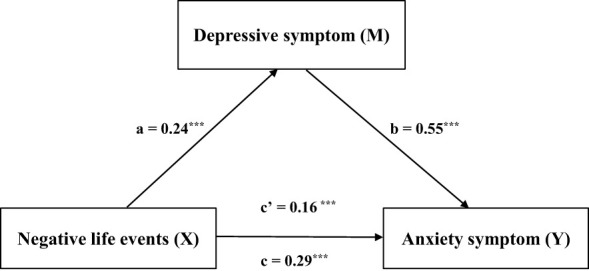
Shows that negative life events influence anxiety symptoms through depressive symptoms, indicating a significant mediation effect. ***p < 0.001.

**Table 6** shows that the overall effect of negative life events on anxiety symptoms was 0.29 (SE = 0.006, 95% CI = 0.28-0.30, t = 46.61, *p* < 0.001). The direct effect was 0.16, accounting for 55.2% of the total effect (SE = 0.005, 95% CI = 0.15-0.17, t = 31.05, *p* < 0.001). The indirect effect was 0.13, accounting for 44.8% of the total effect (SE = 0.005, 95% CI = 0.12-0.14). **Figure 2** shows that negative life events influence anxiety symptoms through depressive symptoms, indicating a significant mediation effect.

**Table 6:** Total, direct, and indirect effects of negative life events (X) on anxiety symptoms (Y) through depressive symptoms (M) (N = 8041).

Effect	Estimate	Boot SE	Total Effect Percentage	Boot 95% CI		t	*p*
				Lower	Upper		
Total effect	0.29	0.006	100%	0.28	0.30	46.61	< 0.001
Direct effect	0.16	0.005	55.2%	0.15	0.17	31.05	< 0.001
Indirect effect	0.13	0.005	44.8%	0.12	0.14	–	–

SE, Standard Error; CI, confidence interval. Based on 5000 bootstrap samples; 95 % Bias Corrected Confidence Interval.

**Figure 3:**
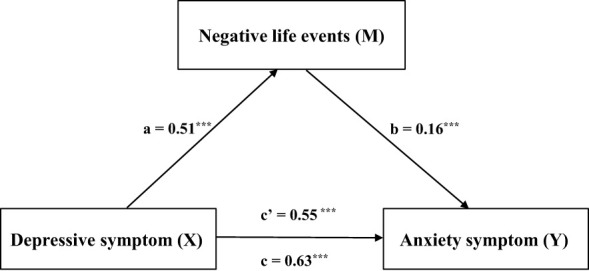
Shows that the effect of depressive symptoms on anxiety symptoms was mediated by negative life events. ***p < 0.001.

**Table 7** shows that the total effect of depressive symptoms on anxiety symptoms was 0.63 (SE = 0.008, 95% CI = 0.62-0.64, t = 83.42, *p* < 0.001), with a direct effect of 0.55, accounting for 87.3% of the total effect (SE = 0.008, 95% CI = 0.53-0.56, t = 71.99, *p* < 0.001), and an indirect effect of 0.08, accounting for 12.7% of the total effect (SE = 0.004, 95% CI = 0.07-0.09). **Figure 3** shows that the effect of depressive symptoms on anxiety symptoms was mediated by negative life events.

**Table 7:** Total, direct, and indirect effects of depressive symptoms (X) on anxiety symptoms (Y) through negative life events (M) (N = 8041).

Effect	Estimate	Boot SE	Total Effect Percentage	Boot 95% CI		t	*p*
				Lower	Upper		
Total effect	0.63	0.008	100%	0.62	0.64	83.42	< 0.001
Direct effect	0.55	0.008	87.3%	0.53	0.56	71.99	< 0.001
Indirect effect	0.08	0.004	12.7%	0.07	0.09	–	–

SE, Standard Error; CI, confidence interval. Based on 5000 bootstrap samples; 95 % Bias Corrected Confidence Interval.

**Figure 4:**
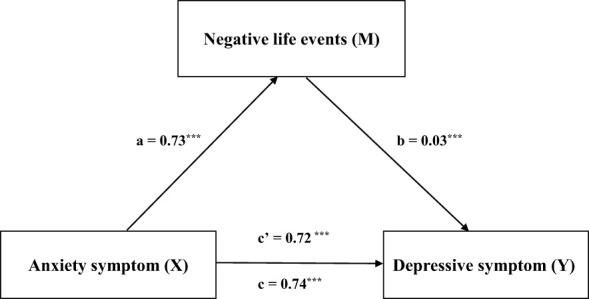
Shows that anxiety symptoms affect depressive symptoms through negative life events. ***p < 0.001.

**Table 8** shows that the total effect of anxiety symptoms on depressive symptoms was 0.74 (SE = 0.009, 95% CI = 0.72-0.75, t = 83.42, *p* < 0.001). The direct effect was 0.72 (SE = 0.01, 95% CI = 0.70-0.74, t = 71.99, *p* < 0.001). The indirect effect was 0.02 (SE = 0.05, 95% CI = 0.01-0.03). **Figure 4** shows that anxiety symptoms affect depressive symptoms through negative life events.

**Table 8:** Total, direct, and indirect effects of anxiety symptoms (X) on depressive symptoms (Y) through negative life events (M) (N = 8041).

Effect	Estimate	Boot SE	Total Effect Percentage	Boot 95% CI		t	*p*
				Lower	Upper		
Total effect	0.74	0.009	100%	0.72	0.75	83.42	< 0.001
Direct effect	0.72	0.01	97.3%	0.70	0.74	71.99	< 0.001
Indirect effect	0.02	0.005	2.7%	0.01	0.03	–	–

SE, Standard Error; C, confidence interval. Based on 5000 bootstrap samples; 95 % Bias Corrected Confidence Interval.

**Figure 5:**
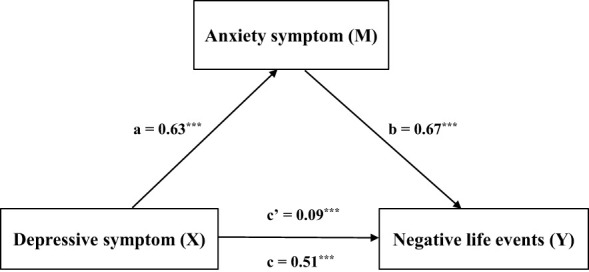
Shows that anxiety symptoms mediate the relationship between depressive symptoms and negative life events. ***p < 0.001.

As shown in **Table 9**, the total effect of negative life events on depressive symptoms through anxiety symptoms was 0.51 (SE = 0.01, 95% CI = 0.48-0.54, t = 33.21, *p* < 0.001). The direct effect was 0.09, accounting for 82.4% (SE = 0.02, 95% CI = 0.05-0.13, t = 4.58, *p* < 0.001). The indirect effect was 0.42, accounting for 17.6% (SE = 0.02, 95% CI = 0.39-0.45). **Figure 5** shows that anxiety symptoms mediate the relationship between depressive symptoms and negative life events.

**Table 9:** Total, direct, and indirect effects of depressive symptoms (X) on negative life events (Y) through anxiety symptoms (M) (N = 8041).

Effect	Estimate	Boot SE	Total Effect Percentage	Boot 95% CI		t	*p*
				Lower	Upper		
Total effect	0.51	0.01	100%	0.48	0.54	33.21	< 0.001
Direct effect	0.09	0.02	82.4%	0.05	0.13	4.58	< 0.001
Indirect effect	0.42	0.02	17.6%	0.39	0.45	–	–

SE, Standard Error; CI, confidence interval. Based on 5000 bootstrap samples; 95 % Bias Corrected Confidence Interval.

**Figure 6:**
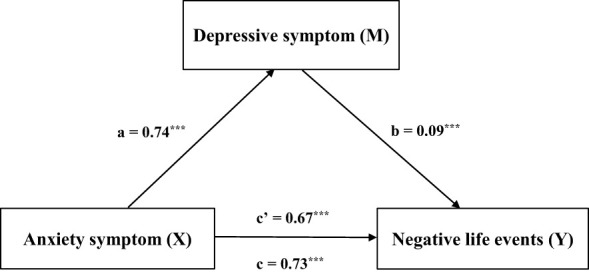
Shows that depressive symptoms play a mediating role in the relationship between anxiety symptoms and negative life events. ***p < 0.001.

As shown in **Table 10**, the total effect of anxiety symptoms on negative life events was 0.73 (SE = 0.02, 95% CI = 0.70-0.76, t = 46.61, *p* < 0.001). The direct effect was 0.67 (90.4%) (SE = 0.02, 95% CI = 0.62-0.71, t = 31.04, *p* < 0.001). The indirect effect was 0.07 (9.6%) (SE = 0.02, 95% CI = 0.03-0.10). **Figure 6** shows that depressive symptoms play a mediating role in the relationship between anxiety symptoms and life events.

**Table 10:** Total, direct, and indirect effects of anxiety symptoms (X) on negative life events (Y) through depressive symptoms (M) (N = 8041).

Effect	Estimate	Boot SE	Total Effect Percentage	Boot 95% CI		t	*p*
				Lower	Upper		
Total effect	0.73	0.02	100%	0.70	0.76	46.61	< 0.001
Direct effect	0.67	0.02	90.4%	0.62	0.71	31.04	< 0.001
Indirect effect	0.07	0.02	9.6%	0.03	0.10	–	–

SE, Standard Error; CI, confidence interval. Based on 5000 bootstrap samples; 95 % Bias Corrected Confidence Interval.

## 4 Discussion

To the best of our knowledge, this study is the first to investigate the relationship between negative life events and psychological problems in children and adolescents, along with their potential mediators. The key findings of this study are as follows: (1) The prevalence of negative life events was 80.52%, while the prevalence of anxiety symptoms and depressive symptoms was 23.38% and 42.57%, respectively. (2) Anxiety symptoms and depressive symptoms were positively correlated with negative life events among children and adolescents; (3) Most importantly, this study found that, among children and adolescents, negative life events, anxiety symptoms, and depressive symptoms each served as mediators in the relationships between the other two factors.

The current study highlights a high prevalence of negative life events (80.52%), anxiety symptoms (23.38%), and depressive symptoms (42.57%) among children and adolescents, findings that align with existing research (28). Prior studies have consistently demonstrated the significant role of negative life events as risk factors for mental health problems in this population. For instance, Zou et al. reported that negative life events are closely associated with increased anxiety and depressive symptoms, particularly in interpersonal and adaptation-related stressors (29). Similarly, Spinhoven et al. observed that childhood adversities, including emotional neglect, contribute substantially to the development of depression and anxiety disorders across the lifespan (30). Moreover, during the COVID-19 pandemic, the prevalence of anxiety and depression was exacerbated by increased exposure to negative life events, as highlighted in Taheri et al., who emphasized the interaction between environmental stressors and genetic predispositions in shaping mental health outcomes (31, 32). These findings underscore the urgency of implementing targeted interventions to mitigate the psychological impact of adverse life events among children and adolescents.

Recent findings demonstrate a significant correlation between negative life events and anxiety and depression in children and adolescents, consistent with prior research. Negative life events such as family conflict, academic pressure, and interpersonal challenges elevate stress levels, impair coping mechanisms, and increase susceptibility to mental health issues like anxiety and depression (29). Adolescents, particularly those prone to rumination, are more vulnerable, with severe depressive symptoms often linked to dwelling on negative experiences (33, 34). The COVID-19 pandemic intensified the impact of negative life events, with increased isolation, disrupted schooling, and parental stress contributing to heightened anxiety and depressive symptoms (35, 36). Vulnerable groups, including those from low socioeconomic backgrounds, were disproportionately affected (37). The interaction between negative life events and mental health outcomes reflects a complex, nonlinear relationship, mediated by psychological resilience, family climate, and coping styles.

Additionally, one of the key findings of this study is the reciprocal mediating relationship among anxiety symptoms, depressive symptoms, and negative life events, demonstrating a bidirectional connection between these factors. This indicates that depressive symptoms play a crucial role in linking negative life events to anxiety symptoms (38). As external stressors, negative life events indirectly influence depressive levels by triggering changes in anxiety symptoms (39, 40). Similarly, these life events can also indirectly affect anxiety levels by altering depressive symptoms (40, 41). This interaction may stem from the psychological stress, social difficulties, and emotional fluctuations caused by life events, which further exacerbate anxiety levels in adolescents (42). The mediating effect highlights the complex relationship between negative life events and anxiety and depression in children and adolescents. The findings suggest that experiencing negative life events significantly increases the risk of anxiety and depression, underscoring the profound impact of such events on the mental health of young individuals.

To address these challenges, targeted interventions are essential. Schools should implement stress-management programs, including peer education and mindfulness training, which have been shown to alleviate anxiety and depressive symptoms (43). Family-based interventions promoting open communication and reducing unrealistic expectations can mitigate stress. For adolescents affected by the pandemic, online counseling and cognitive-behavioral therapy (CBT) are effective strategies (44, 45). Additionally, structured physical activity programs can enhance resilience and reduce mental health symptoms (31, 32). Policymakers should prioritize low-threshold mental health support systems, ensuring accessibility for at-risk groups. Long-term monitoring and large-scale studies are critical for developing evidence-based interventions tailored to the developmental needs of children and adolescents (46).

This study has several limitations that warrant discussion. Firstly, the cross-sectional design limits the ability to infer causality, as it only captures associations between variables at a single point in time. While this design was selected due to its efficiency and feasibility during the COVID-19 pandemic, it restricts the exploration of dynamic changes and causal pathways. Future research should adopt longitudinal designs to track changes over time and establish more robust causal relationships between variables, such as the impact of negative life events on mental health outcomes. Secondly, the study relies on self-reported measures, including the Adolescent Life Events Scale, SAS, and SDS, which are susceptible to response bias and social desirability bias. Although these tools are widely validated and suitable for assessing subjective experiences, they may not fully capture objective realities. To address this limitation, future studies could incorporate multi-method approaches, such as behavioral observations or physiological assessments, to complement self-reported data and reduce potential biases. Thirdly, the use of convenience sampling may reduce the generalizability of the findings to broader populations. This method was chosen due to logistical constraints and the need for rapid data collection; however, it introduces selection bias and limits the representativeness of the sample. Future research should aim to include more diverse and representative samples using random sampling techniques to enhance the reliability and applicability of the results. Fourthly, the study emphasizes correlations and mediation analyses but lacks experimental or intervention-based evidence to confirm the directional or causal relationships among variables. While the findings provide valuable preliminary insights into potential pathways and mechanisms, future studies should incorporate experimental designs or intervention-based approaches to validate and expand upon these results. Fifthly, this study did not fully explore potential mediators or moderators such as resilience, family support, and coping mechanisms. Future research will focus on these factors to further investigate their roles in the relationship between negative life events, anxiety, and depression, providing a more comprehensive understanding of adolescent mental health. Finally, the study does not delve into the mechanisms by which different types of negative life events influence anxiety and depressive symptoms. Understanding these mechanisms is crucial for developing targeted interventions. Future research should explore these pathways to identify specific mediators and moderators of the relationship between negative life events and mental health outcomes, thereby advancing theoretical frameworks and practical applications in this field.

## 5 Conclusion

In conclusion, this study highlights the complex interplay between anxiety, depression, and negative life events in children and adolescents, with bidirectional mediating relationships emphasizing the pathways through which negative life events impact mental health. These findings provide a scientific foundation for targeted interventions, including early psychological support, family- and school-based programs, and resilience-building strategies, to help mitigate the adverse effects of negative life events and enhance mental well-being.

## Data Availability

The raw data supporting the conclusions of this article will be made available by the authors, without undue reservation.
